# Mechanical Properties of Hybrid PVA–Natural Curaua Fiber Composites

**DOI:** 10.3390/ma15082808

**Published:** 2022-04-11

**Authors:** Bartosz Zukowski, Yasmim Gabriela dos Santos Mendonça, Igor José Koury Tavares, Romildo Dias Toledo Filho

**Affiliations:** 1 Department of Civil Engineering, COPPE, Universidade Federal do Rio de Janeiro, Rio de Janeiro 21941-972, RJ, Brazil; bzukowski87@gmail.com (B.Z.); yasmim@numats.coc.ufrj.br (Y.G.d.S.M.); 2 Department of Civil Engineering, Universidade Federal do Pará—UFPA, Av. Perimetral 2651, Belém 66077-830, PA, Brazil; igorkoury@gmail.com

**Keywords:** SHCC, curaua fiber, PVA, hybrid composite, natural fiber

## Abstract

This work presents the experimental study of hybrid cement-based composites with polyvinyl alcohol fiber (PVA) and alkali-treated, short, natural curaua fiber. The objective of this research is to develop composites reinforced with PVA and curaua fiber to present strain-hardening behavior with average crack width control. To achieve this objective, three groups of composites were investigated. The first group had only PVA fiber in volumes of 0.5, 1, and 2%. The composite with 2% PVA fiber was the only one with strain-hardening and crack width control. The second group had 0.5% PVA fiber and volume fractions of 2, 2.5, and 3% curaua fiber, and presented only deflection-hardening behavior. The third group had 1% PVA and volumes of 1, 1.5, and 2% curaua fiber, and presented strain-hardening behavior. Based on the results, the hybrid combination of 1% PVA and 1.5% curaua was the optimal mixture as it presented strain-hardening behavior and crack width control, with a lower volume of synthetic PVA fiber. Additionally, compressive strength and mix workability were calculated for the investigated composites for comparison.

## 1. Introduction

Strain-hardening cement-based composites (SHCC) are composites reinforced with synthetic fiber, which, after reaching first crack strength, present multiple-cracking-behavior and maintain resistance along with new fine crack formation [1]. The design process of SHCC is based on the micromechanical model [2] where matrix fracture properties (first crack stress), fiber–matrix interaction (bond energy and frictional tension), and the fiber characteristics (length, diameter, and elasticity modulus) are combined to predict the critical fiber volume needed for strain-hardening to occur. Due to its high strain capacity, SHCC has found application in structural elements like bridge decks [3], columns and beams [4], and building dampers [5], which can suffer deformation due to service loads. SHCC is also used in water channels [6] and other hydraulic structures [7,8] because of its capacity to form multiple thin crack patterns that could prevent leakage and present self-healing properties [9].

### 1.1. Curaua Fiber

**Figure 1:**
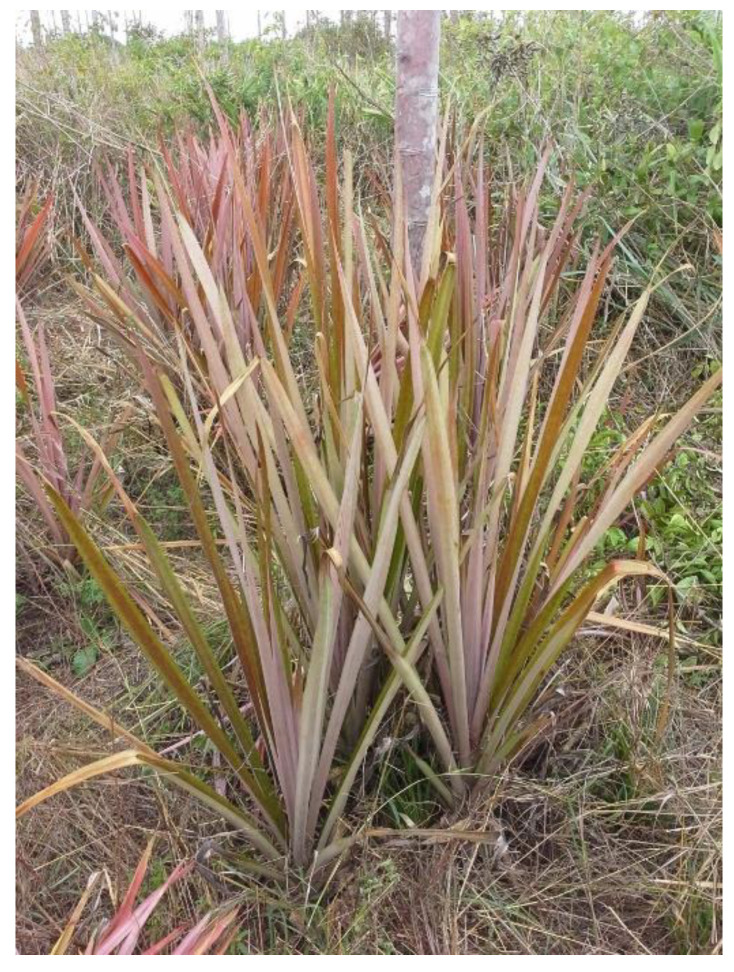
Curaua plant with paricá tree plantation.

The curaua (or curauá), *Ananas comosus* var. *erectifolius* (description given by L.B. Smith), is a type of pineapple plant cultivated for the fiber from its leaves in hot and humid climates, mostly in the Amazon region of Brazil. It can be cultivated on sandy and sandy–clay soils. It has been seen that curaua can grow along the paricá tree (Figure 1) and can be used in reforestation.

**Figure 2:**
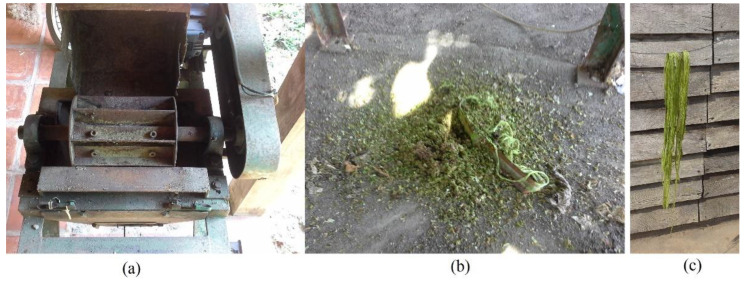
Curaua fiber extraction. (**a**) Machine used to separate fiber from leaves. (**b**) Mucilage residue after fiber extraction. (**c**) Curaua fiber drying in the sun.

The fibers are separated from the leaves by a mechanical process [10], then washed and dried for impurities and residue removal [11], as illustrated in Figure 2. The washing process is important for fiber durability because residues can cause fungus growth on the fibers’ surface and reduce their life span. After washing, the fibers are dried for 48 h in natural conditions under direct sunlight at a temperature and humidity specific to the location of the plantation, and then fibers are tied into bundles and are ready for use.

The motivation behind curaua fiber use is in the low initial capital required for start-up production, its simple cultivation methods, and its simple fiber extraction process. All of these aspects create the possibility to improve the economic conditions of the northern Amazonian states of Brazil [12]. The demand for curaua has risen since its application in the automotive industry as an ecological alternative to glass fiber. Because of that movement, new materials are being investigated which could incorporate curaua fiber in their structures as a biocomposite [13] or cement-based composite [14]. Curaua fiber is considered a high-performance fiber due to its high tensile resistance, which is above 600 MPa, and its high Young’s modulus, which is above 38 GPa [15].

### 1.2. SHCC with Curaua Fiber

After synthetic fibers, such as polyvinyl alcohol fiber (PVA), polyethylene fiber (PE), and polypropylene fiber (PP), natural fibers have found applications in SHCC composites. A comparison of the properties of these fibers and the curaua fiber is shown in Table 1. It can be seen that curaua fiber has a diameter, density, Young’s modulus, and failure strain similar to the synthetic fibers. The design approach presented 10–20 mm curaua fiber, which, after washing cycles and combing, was implemented into a cement matrix with an admixture of coupling agent (vinyltriethoxysilane (VS)) to improve the fiber–matrix bond. The addition of 4.4% per volume fibers with 1% coupling agent presented a multiple-cracking pattern with strain-hardening behavior and a strain capacity of 0.8% [16]. The micromechanical design indicated that the 4% per volume of 20 mm alkali-treated curaua fibers was sufficient for strain-hardening to occur [17]. The strain capacity of the composite increased with fiber length, and for 40 mm, the composites presented a 0.8% strain capacity with a slight tensile strength improvement and new crack formation. The composites exposed to natural weathering did not maintain strain-hardening properties due to fiber–matrix bond deterioration. The lower fiber–matrix bond was not sufficient to transfer the load prior to new crack formation. However, the curaua fiber remained in the composite and did not petrify in the cement matrix. Moreover, the fibers extracted from the composite presented resistance to tensile load, which indicated a favorable durability of the natural fibers in the matrix durability [18].

### 1.3. Hybrid SHCC

Successful trials have been reported for hybrid, strain-hardening composites that incorporate different fibers and that benefit from the combined fiber properties. The combination of steel (0.5%) and PVA (1.5%) fibers in a composite improved fire resistance and reduced explosive spalling when exposed to high temperatures. The admixture of steel fiber prevented the composite from brittle failure after exposure to elevated temperatures above the PVA melting point (225 °C) [21]. The incorporation of PVA and steel fibers into a micromechanical model predicted that 2% of PVA with 0.5% of steel fiber composition would yield an optimal tensile strain capacity [22,23]. Economic and environmental benefits are further reasons for SHCC hybridization. The trial of PVA (1%) with PET (1%) fibers was recommended as a combination for impact resistance composites. The incorporation of 1% of PET fibers provided a similar impact resistance to 2% PVA-SHCC-only composites, but at lower cost [24]. Moreover, the hybrid solution still presents strain-hardening properties and crack control [25]. PVA fibers have also been combined with PE fibers. The hybrid PVA–PE composites presented strain-hardening behavior, and when subjected to 300 cycles of rapid freezing and thawing, they maintained this behavior, but with lower strain capacity [26]. A PE and steel fiber SHCC hybrid has found an application in lap-splicing reinforcing bars, where bond strength and ductility were improved in comparison to a non-hybrid SHCC [27].

The literature reveals many past studies on SHCC with hybrid synthetic fibers; however, there are no studies that address this issue by combining synthetic fibers and natural fibers. This is the main motivation of this study. This work presents the results of PVA fiber partial substitution by alkali-treated, natural curaua fiber of 20 mm length. The fiber compositions were chosen to investigate the possibility to achieve strain-hardening behavior with the lowest PVA fiber content, with a high strain capacity original to PVA-only SHCC. The benefits of the hybridization can include cost reduction and a lower environmental impact due to the incorporation of natural fiber in the composite. The composites reinforced with curaua fiber only presented a durability challenge in maintaining strain-hardening behavior when exposed to natural weathering. The lack of desired behavior was related to the deterioration of the fiber–matrix interaction [18]. The fiber reinforcement hybridization can be a solution for the durability issues discovered for SHCC with curaua only. Due to a higher volume content than typical SHCC (above 2% per volume), the investigation of the workability of the mixes was carried out to provide more information on hybrid compositions.

## 2. Materials and Methods

### 2.1. Materials

#### 2.1.1. Matrix

The matrix composition used to make the composites included Portland Cement CP II F-32, fly ash, and metakaolin from Metacaulim do Brasil, São Paulo, SP, Brazil in proportions of 1:0.5:0.4 (cementitious materials, sand, and water/cementitious materials, respectively) and was 33% cement, 27% metakaolin, and 40% fly ash. The fly ash and the metakaolin were used to ensure a calcium-free hydroxide matrix, since calcium hydroxide is harmful for natural fiber durability [28]. To improve workability, a 2.9% admixture of Glenium 51 (31% of the solids) was added, as was 1% of the viscosity modifier Rheomac UW 410 (both manufactured by BASF São Paulo, SP, Brazil).

Sand of a diameter less than 212 µm was used for casting. The matrix composition (Table 2) proved to be free of calcium hydroxide and was favorable for natural fiber durability [18]. The chemical composition of the cementitious materials used in the study are presented in Table 3.

The cementitious paste composed of water and cementitious materials was aged for 28, 60, and 90 days, and underwent thermal analysis using the simultaneous DSC-TGA model SDT Q600 from TA Instruments. The equipment operated with a flow temperature of 1 °C/min with a temperature of approximately 10 °C in a nitrogen atmosphere with an ambient air flow rate of 100 mL/min.

#### 2.1.2. PVA Fiber

The PVA REC 15 fiber used for the composites was provided by the Kuraray company (Tokyo, Japan). The fiber length was 12 mm, with a diameter of 0.04 mm. According to the producer, the fibers presented a tensile strength of 1600 MPa, a Young’s modulus of 41 GPa, and a 6% deformation capacity [29].

#### 2.1.3. Curaua Fiber

The curaua fibers were provided in bundles by Pematec (Santarem, Para, Brazil). Firstly, fibers were submitted to three cycles of washing in hot water (80 °C) for 3 h, followed by 24 h of drying at 40 °C to remove residues and impurities from the surface, as well as to reduce the area of the cross-section [30]. Secondly, the washed fiber bundles were combed for fiber separation and cut into 20 mm lengths. Thirdly, the short, separated fibers were immersed in a 1% alkali solution of calcium hydroxide Ca(OH)_2_ for 60 min and then dried for 24 h at 40 °C. The curauá fibers used in this study presented a tensile strength of 697 MPa, Young’s modulus of 39 GPa, fiber length of 20 mm, and diameter of 77 mm. This last stage, called alkaline treatment, causes a decrease in the degree of polymerization and crystallinity, as well as a separation of structural linkages between lignin and cellulose and a partial disintegration of the lignin structure [31]. As a result, the treatment removes some hemicellulose and lignin from the fiber wall and increases surface roughness by calcium deposition, which improves the fiber–matrix bond properties [17].

#### 2.1.4. Composites

The composites used in the bending and tensile tests were cast in forms of square plates measuring 450 mm × 450 mm × 20 mm, which later were cut into smaller plates measuring 400 mm × 60 mm × 20 mm to be used for tensile and flexural testing using a circular saw blade cutting machine. The procedure of casting was based on a similar procedure used for SHCC with alkali-treated curaua fiber [17]. The procedure began by mixing all dry ingredients (cement, fly ash, metakaolin, sand, and viscosity modifier), followed by the addition of water and superplasticizer, similar to the standard ECC mixing sequence [32]. At the end, fibers were added to the mix, maintaining the maximum separation possible, which was achieved by manual dosage. The procedure of composite casting was divided into following steps: (i) 30 s of dry ingredient mixing at a speed of 125 rpm; (ii) 30 s of admixture of water during mixing at a speed of 125 rpm; (iii) 60 s of admixture of 50% superplasticizer at a speed of 220 rpm; (iv) 30 s of adding 50% superplasticizer at a reduced speed of 125 rpm; (v) 60 s of mixing at a speed of 220 rpm; (vi) 60 s of rest; (vii) 120 s of fiber addition at a speed of 125 rpm; (viii) 60 s of mixing at a speed of 220 rpm; (ix) casting into molds; and (x) 30 s on a vibration table. The composites were cast into plastic formwork and stored for 48 h before being removed from the form. Next, the specimens were stored for up to 28 days in an environmental chamber at 21 °C to provide humid conditions (relative humidity above 90%).

The three groups of composites were cast. The first group of composites was reinforced with PVA fiber only and had a fiber content of 0.5%, 1%, 2% per volume. The second group included the hybrid composites with 0.5% PVA fiber and 2%, 2.5%, and 3% curaua fiber. The third group included the hybrid composites with 1% PVA and 1%, 1.5%, and 2% curaua fiber. Table 4 presents the three groups of casted composites with the corresponding fiber volume content and hybrid combinations. The criteria used for the volume selection was based on the objective of creating a hybrid PVA–curaua fiber composite with strain-hardening behavior and a lower volume of PVA fiber. Along with hybrid combinations, the composites with 0.5% and 1% PVA fiber were prepared and tested.

It was reported that for the volume content of 4% curaua fiber for a given matrix, strain-hardening occurred [17], and so the hybrid compositions were designed to use a total fiber amount of less than 4%. The fiber dosage of the hybrid composites was chosen to simplify the process of design.

### 2.2. Testing Methods

#### 2.2.1. Compressive Strength

The compressive strength of the composites was verified on cylindrical specimens (10 cm × 5 cm) after 28 days of curing. The tests were carried out according to ABNT NBR 5739 at a temperature of 22 ± 2 °C, using a Shimadzu model UH-F with a load cell of 1 kN and deformation velocity of 0.1 mm/min.

#### 2.2.2. Flexural Strength

**Figure 3:**
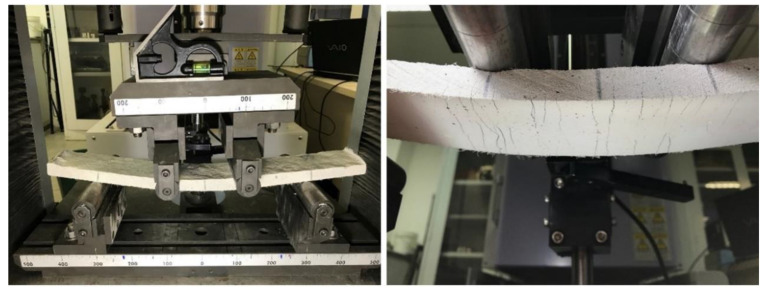
Flexural strength test. (**left**) Test set-up. (**right**) Detail of a deformed specimen with multiple bending cracks.

The flexural strength of the composites (Figure 3) was verified by a four point bending test on plates with dimensions of 400 mm × 60 mm × 20 mm (carried out using a Shimadzu AGX model with a load cell of 1 kN and 0.3 mm/min velocity). The span between supports was equal to 300 mm. The LVDT was used in the central point of the plate for deformation measurements. The first crack flexural strength and maximum flexural strength were calculated according to Equation (1):

(1) $$\sigma_{flexural}=\frac{FL}{bd^{2}}$$

where *F* is the applied force, *L* is the span between the supports, *b* is the specimen width, and *d* is the specimen thickness.

#### 2.2.3. Tensile Strength and Crack Width Analysis

**Figure 4:**
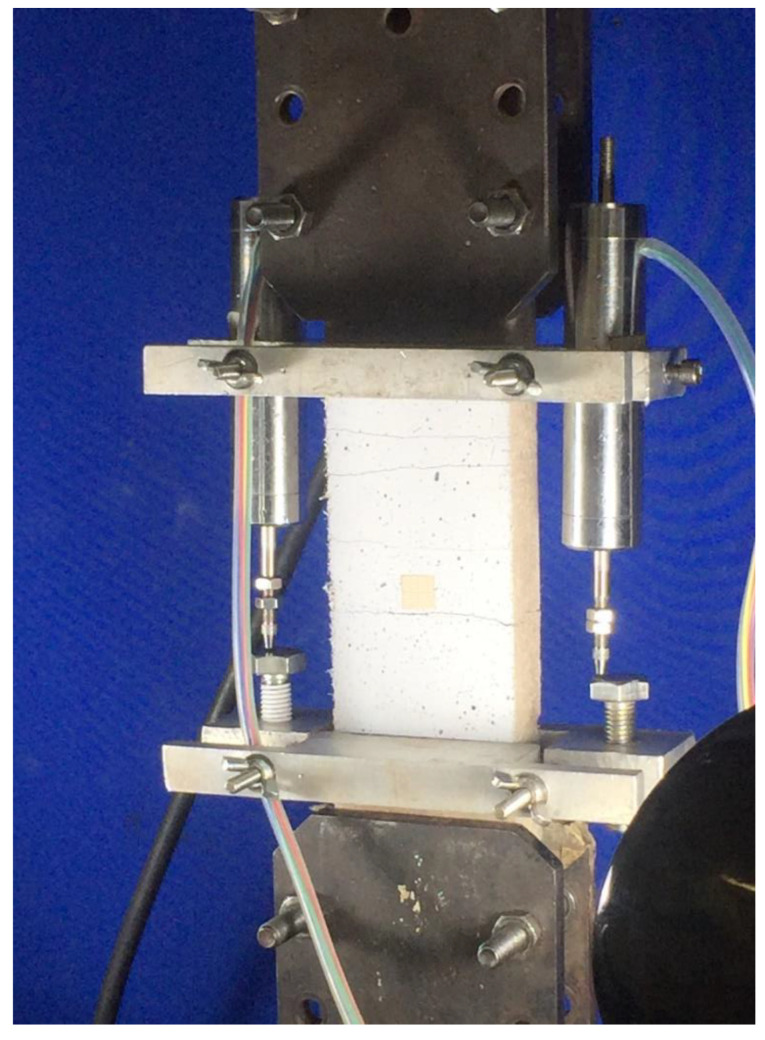
Tensile strength test setup.

The tensile strength of the composites (Figure 4) was verified on plate specimens measuring 400 mm × 60 mm × 20 mm. The specimens were mounted in steel grips to provide 100 mm of clear span for tension analysis. The tests were carried out using a Shimadzu AGX model with a load cell of 1 kN and 0.1 mm/min velocity. The composite’s deformation was measured simultaneously by two LVDTs located by the sides of the specimen. Additional camera footage with intervals of 30 s during tests was used for crack verification. The average crack width was calculated based on an indirect method where the specimen’s deformation was divided by the number of cracks formed. The results were presented along a stress–strain curve as an average crack width tendency line.

#### 2.2.4. Matrix Workability

Due to the high fiber volume content of hybrid composites, up to 3–3.5% of the flow table test was applied for evaluation of the mix workability, according to NBR 13,276 [33].

## 3. Results and Discussion

### 3.1. Thermal Analysis of the Cementitious Paste

**Figure 5:**
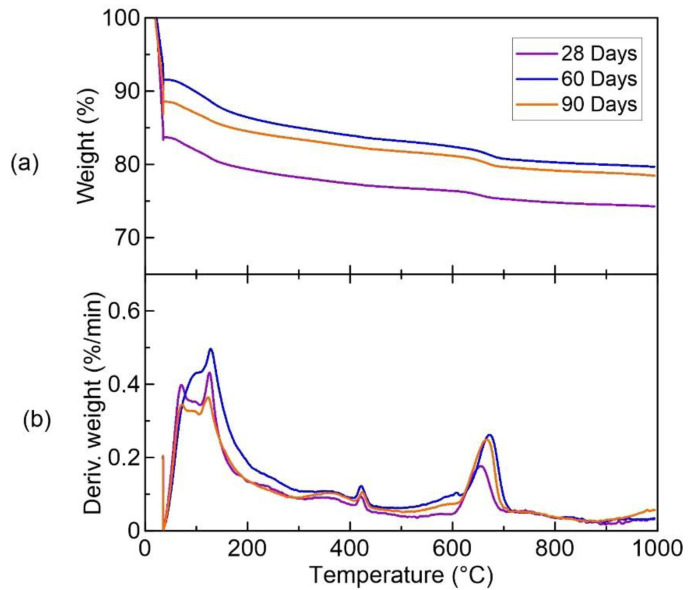
Thermal analysis of the paste at 28, 60, and 90 days: (**a**) weight versus temperature; (**b**) derivate of weight versus temperature.

The cementitious paste composed of water and cementitious materials was aged for 28, 60, and 90 days (Figure 5) and underwent thermal analysis that proved that the matrix was free of calcium hydroxide (CH) after 28 days of hydration. For all ages, the content of CH was in the range of 0.2–0.3%. The dihydroxylation of the calcium hydroxide was calculated at a temperature of 400–450 °C, according to the literature recommendation [34].

### 3.2. Composites

The results of the tests performed on the specimens are presented in Table 5. From the PVA composites, it can be observed that with increased amounts of fiber, the flexural strength, the tensile strength, and the tensile maximum strength increased proportionally. However, the increments of fiber decreased the compressive strength. The PVA composite of 2% fibers presented less compressive strength, but higher flexural strength and tensile strength at first crack, as well as tensile maximum strength, compared to other PVA composites. The hybrid composites of 1% PVA and 1.5% curaua presented a better flexural strength and tensile maximum strength between the hybrid composites. Comparing the composites PVA 2 and HYB 1–1.5, it is possible to affirm that there is no considerable variation between their performance related to compressive strength, flexural strength at first crack, tensile strength at first crack, and tensile maximum strength. In addition, the PVA 2 and HYB 1–1.5 composites presented a higher tensile maximum strength compared with the other composites.

#### 3.2.1. Mix Workability

**Figure 6:**
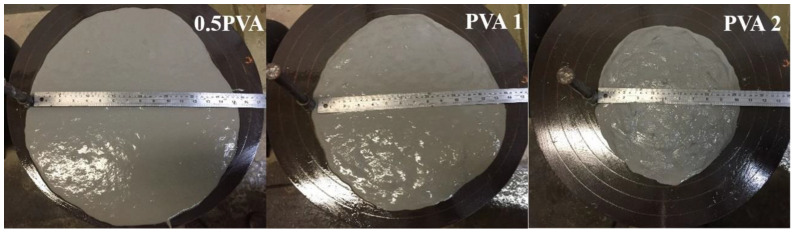
Flow table of the composites PVA 0.5, PVA 1, and PVA 2.

**Figure 7:**
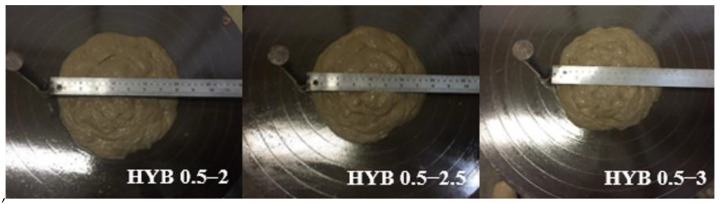
Flow table of the composites HYB 0.5–2, HYB 0.5–2.5, and HYB 0.5–3.

**Figure 8:**
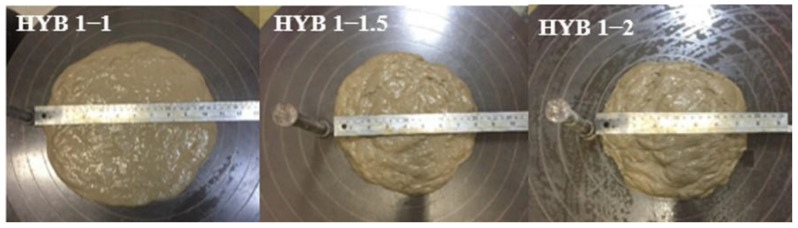
Flow table of the composites HYB 1–1, HYB 1–1.5, and HYB 1–2.

The mix workability was mostly reduced by the volume of the fiber applied. Figure 6, Figure 7 and Figure 8 illustrate the results of table flow for the composites with only PVA and the hybrids of 0.5% PVA and 1.0% PVA, respectively. The increased fiber volume mixture reduced flow ability, which can influence the casting and uniform fiber distribution along with the element. The composites reinforced with only 2% PVA fiber presented a table flow value of 260 mm. The hybrid composition with a similar table flow value of 265 mm had a similar total fiber volume of 1% of PVA and 1% of curaua. The composites with a total fiber volume equal to 2.5% presented similar table flow values independent of the percentage of PVA fiber. Thus, the hybrids HYB 0.5–2 and HYB 1–1.5 presented table flow values of 200 and 210 mm.

All mixtures presented good casting capacity, especially those with flow table values above 195 mm, with good mouldability.

#### 3.2.2. Compressive Strength

For the composites with only PVA fiber, there is a visible pattern for compressive strength reduction related to increased fiber volume. For the specimens with 0.5% fiber, the highest average compressive strength reported was equal to 45.53 MPa. Doubling the amount of fiber reduced the compressive strength to 41.46 MPa for 1% fiber per volume. This pattern continues for 2% PVA fiber with 35.86 MPa average compressive strength.

**Figure 9:**
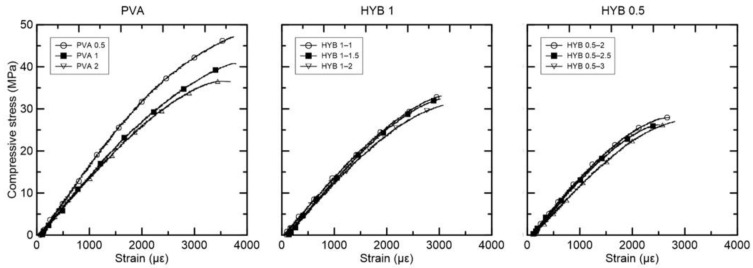
Compressive stress versus strain curves for the composites reinforced with PVA, HYB 1, and HYB 0.5.

Figure 9 presents the compressive stress versus strain curves for the composites reinforced with PVA, HYB 1, and HYB 0.5. In the case of hybrid composites, all compositions presented lower compressive strength in comparison to only-PVA fiber composite, which is related to higher fiber volume in total. In the case of hybrid compositions, the total fiber volume was crucial for average compressive strength. Thus, the hybrid compositions with 0.5% PVA fiber presented lower values of 29.59 MPa for 2.5%, 29.74 MPa for 3%, and 26.26 MPa for 3.5% total fiber volume. The hybrid compositions with 1% PVA presented average compressive strength values of 32.24 MPa for 2%, 31.93 MPa for 2.5%, and 29.77 MPa for 3% total fiber volume. In addition, it can be noted that the composite HYB 1–1.5 presented higher compressive strength compared to HYB 0.5–2%. This suggests that the effect of PVA on strength is different for curaua fiber, considering that the composite with 1% curaua presented lower compressive strength than the composite with 0.5% PVA, though both had the same total fiber amount.

#### 3.2.3. Flexural Strength

**Figure 10:**
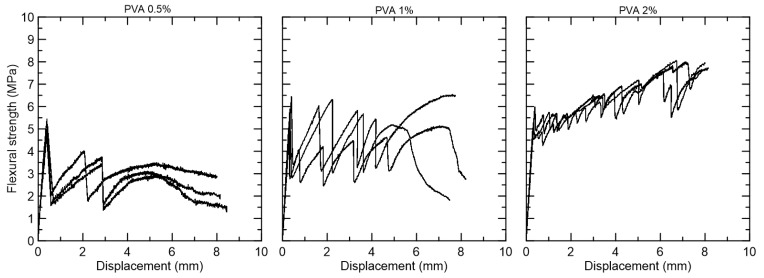
Flexural strength versus displacement curves for the composites reinforced with 0.5%, 1%, and 2% PVA fiber.

The PVA composites presented an average flexural strength at first crack above 5 MPa. The specimens with 0.5% PVA fiber presented deflection-softening behavior, with only two cracks formed. The specimens with 1% PVA fiber also presented deflection-softening, but with more cracks during the deformation process. The specimens with 2% PVA fiber presented deflection-hardening behavior, with multiple fine cracks formed during the deformation process (Figure 10).

**Figure 11:**
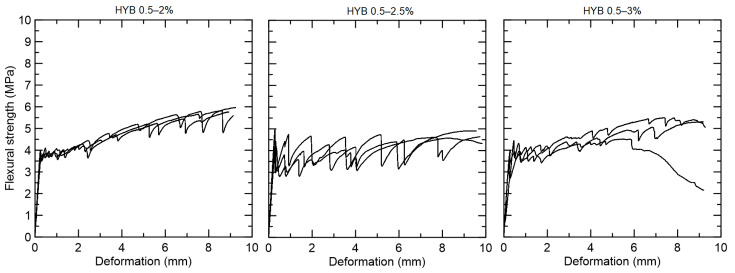
Flexural strength versus displacement curves for the hybrid composites reinforced with 0.5% PVA and 2%, 2.5%, and 3% curaua fiber.

The hybrid compositions with 0.5% PVA and 2%, 2.5%, and 3% curaua fiber presented fine cracks under bending during the deformation process (Figure 11). The specimens presented deflection-hardening behavior with a flexural strength at first crack equal to 3.90 MPa for HYB 0.5–2, 4.49 MPa for HYB 0.5–2.5, and 3.92 MPa for HYB 0.5–3. All groups presented lower flexural strength at the first crack in comparison with only-PVA 0.5% fiber composite (5.18 MPa).

**Figure 12:**
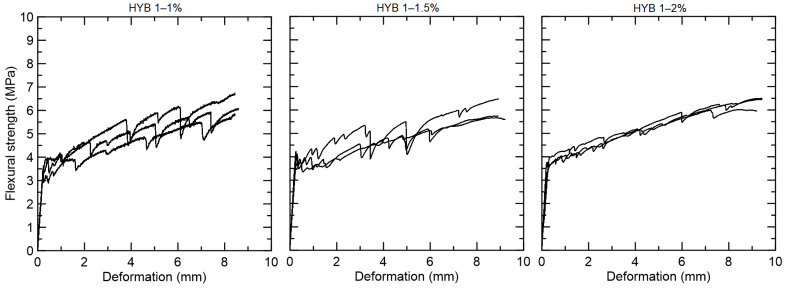
Flexural strength versus displacement curves for the hybrid composites reinforced with 1% PVA and 1%, 1.5%, and 2% curaua fiber.

The hybrid compositions with 1% PVA and 1%, 1.5%, and 2% curaua fiber presented deflection-hardening behavior and fine crack formation (Figure 12). The flexural strength at the first crack was equal to 3.56 MPa for HYB 1–1, 4.01 MPa for HYB 1–1.5, and 3.96 MPa for HYB 1–2. The flexural strength at first crack was lower in comparison with only-PVA 1% fiber (5.77 MPa), with a post-cracking strength superior to 6 MPa for all mixtures tested.

All hybrid composites presented lower flexural strength at the first crack in comparison to the corresponding composites reinforced only with PVA fiber. This is related to the increased total fiber volume in the composite and different fiber structures. PVA fiber is a synthetic fiber with a solid and smaller cross-section area in comparison to curaua. Since the bridging is not activated before the first crack appears, natural fiber presence can disturb the matrix integrity and lower the flexural strength at the first crack. After cracking, the higher volume of fibers can successfully bridge the cracked matrix and provide multiple crack formation along with deflection-hardening. In the case of composites reinforced only with 0.5% or 1% of PVA fiber, the volume (or the number of fibers per crack) is not sufficient to sustain the cracking load and deflection-softening occurs.

#### 3.2.4. Tensile Strength

**Figure 13:**
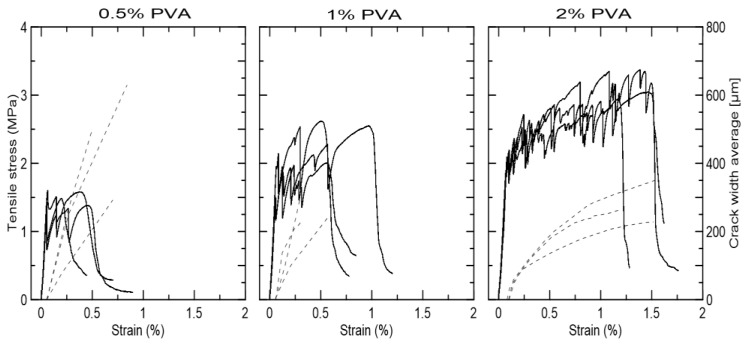
Tensile strength (continues line) versus strain curves and average crack width (dotted line) for the composites reinforced with 0.5%, 1%, and 2% PVA fiber.

The performance of the composites with only PVA fiber was significantly impacted by the fiber volume. The composites with only 0.5% PVA fiber did not present strain-hardening behavior or crack width control. The first crack occurred at 1.50 MPa and no fine cracks were formed (Figure 13). The specimens with 1% PVA fiber presented the first crack at 1.65 MPa and a maximum tensile strength of 2.39 MPa. The strain-hardening behavior occurred up to strain of 0.5%, but with no crack width control. The specimens with 2% PVA fiber cracked at 1.95 MPa and presented strain-hardening behavior up to 3.12 MPa, and 1.25–1.5% strain with multiple fine cracks of below 350 µm average width.

**Figure 14:**
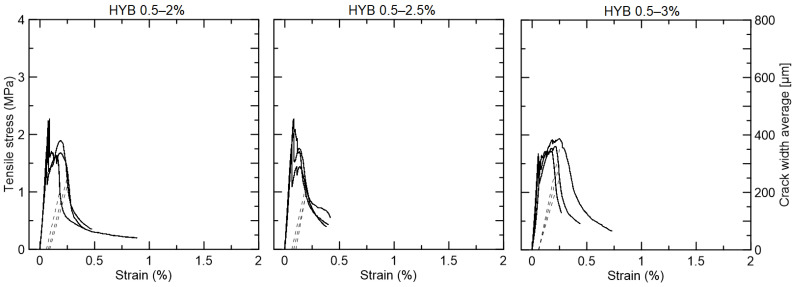
Tensile strength (continues line) versus strain curves and average crack width (dotted line) for the hybrid composites reinforced with 0.5% PVA and 2%, 2.5%, and 3% curaua fiber.

The hybrid compositions with 0.5% PVA and 2% or 2.5% curaua did not present strain-hardening behavior and the first crack strength was the maximum tensile strength achieved, equal to 2.07 MPa for the former and 2.06 MPa for the latter (Figure 14). The hybrid composition with 3% curaua presented a slight increase in tensile strength after the first crack at 1.63 MPa up to 1.82 MPa, but without any crack width control and a maximum strain in the range of 0.3%.

**Figure 15:**
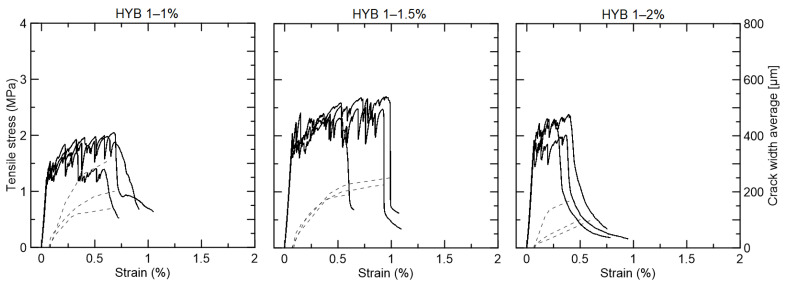
Tensile strength (continues line) versus strain curves and average crack width (dotted line) for the hybrid composites reinforced with 1% PVA and 1%, 1.5%, and 2% curaua fiber.

The hybrid composition with 1% PVA fiber presented strain-hardening behavior with modest strain capacity (Figure 15). The group with 1% curaua cracked at 1.39 MPa and improved up to 1.91 MPa, but without crack width control. The most promising group was HYB 1–1.5, which was 1% PVA and 1.5% curaua. This group presented strain-hardening behavior, with the first crack strength at 1.92 MPa and a maximum strength reaching 2.53 MPa. Moreover, the average crack width was controlled below 250 µm, up to 0.6–1% of strain capacity. The hybrid composition with 2% curaua also presented strain-hardening behavior from 1.84 MPa to 2.23 MPa, but with a strain capacity of lower than 0.5%.

## 4. Conclusions

The study presented the results of workability, compressive strength, four-point bending, and tensile strength tests for specimens with only PVA and PVA–natural curaua fiber composites. The conclusions based on the results are:

- Workability of the mix is mostly influenced by the total fiber volume, and with a higher fiber volume, there are lower table flow spread values. All of the studied compositions presented good casting capacity.
- Compressive strength is influenced by total fiber volume, and the higher the total fiber volume, the lower the compressive strength, especially for high volume fractions of vegetable fibers.
- Composites reinforced with 2% PVA fiber presented deflection-hardening behavior and strain-hardening behavior. Similar behavior was achieved with a combination of 1% PVA fiber and 1.5% curaua fiber. The hybrid composites presented lower strain capacity in comparison to only-PVA fiber composites.
- The application of short curaua fiber, along PVA fiber, creates an opportunity to achieve the strain-hardening behavior of composites with a lower volume of PVA fiber and similar workability.

## Figures and Tables

**Table 1:** Properties of the synthetic fibers and the natural curaua fiber.

Fiber	Diameter (µm)	Density (g/cm^3^)	Tensile Strength (MPa)	Young’s Modulus (GPa)	Failure Strain (%)	Reference
PVA	1.30	1.30	600–2500	5–50	6–17	[19]
PE	7–150	0.96	200–300	5–6	3–4	[19]
PP	20–70	0.91	300–700	3.5–11	15–25	[19]
Curaua	90	1.29	605	23	2.5	[20]

**Table 2:** Matrix composition for 1 m^3^.

	Cement	Metakaolin	Fly Ash	Sand	Water	Glenium	Rheomac
kg/m^3^	361.85	289.85	434.22	542.78	417.97	24.67	0.87

**Table 3:** Chemical composition in % of cement (CP II F-32), fly ash (PozoFly), and metakaolin (Metacaulim HP Ultra).

Oxide	Cement	Metakaolin	Fly Ash
Al_2_O_3_	3.78	41.69	28.24
SiO_2_	13.64	51.85	51.58
Fe_2_O_3_	-	1.91	-
TiO_2_	0.30	1.38	1.3
SO_3_	3.97	1.09	1.51
K_2_O	0.39	1.89	3.39
ZrO_2_	-	0.03	-
BaO	-	-	-
Cr_2_O_3_	-	0.01	-
MnO	0.06	-	1.51
ZnO	0.05	-	-
SrO	0.30	-	-
CaO	73.09	-	1.94
CuO	0.02	-	-

**Table 4:** Composite fiber content per volume (%).

Composite	PVA	Curaua	Total
PVA 0.5	0.5	0	0.5
PVA 1	1	0	1
PVA 2	2	0	2
HYB 0.5–2	0.5	2	2.5
HYB 0.5–2.5	0.5	2.5	3
HYB 0.5–3	0.5	3	3.5
HYB 1–1	1	1	2
HYB 1–1.5	1	1.5	2.5
HYB 1–2	1	2	3

**Table 5:** Collected results for PVA-only and hybrid composites.

Composite	Compressive Strength [MPa]	Flexural Strength at First Crack [MPa]	Tensile Strength at First Crack [MPa]	Tensile Maximum Strength [MPa]	Table Flow [mm]
PVA 0.5	45.53 ± 2.04	5.18 ± 0.24	1.50 ± 0.09	1.50 ± 0.09	385
PVA 1	41.46 ± 1.18	5.77 ± 0.74	1.65 ± 0.26	2.39 ± 0.34	340
PVA 2	35.86 ± 1.76	5.72 ± 0.24	1.95 ± 0.12	3.12 ± 0.23	260
HYB 0.5–2	29.59 ± 1.22	3.90 ± 0.04	2.07 ± 0.32	2.07 ± 0.32	200
HYB 0.5–2.5	29.74 ± 0.85	4.49 ± 0.42	2.06 ± 0.23	2.06 ± 0.23	190
HYB 0.5–3	26.26 ± 0.84	3.92 ± 0.55	1.63 ± 0.04	1.82± 0.11	185
HYB 1–1	32.24 ± 0.74	3.56 ± 0.29	1.39 ± 0.18	1.91 ± 0.18	265
HYB 1–1.5	31.93 ± 1.21	4.01 ± 0.26	1.92 ± 0.10	2.53 ± 0.20	210
HYB 1–2	29.77 ± 192	3.96 ± 0.20	1.84 ± 0.06	2.23 ± 0.19	195

## Data Availability

Not applicable.
